# The Relationship between Selected Factors (Temperament, Bipolar Traits, Sleep Quality, Severity of Addiction) and Depressive Symptoms in Alcohol-Dependent Men

**DOI:** 10.3390/ijerph20054072

**Published:** 2023-02-24

**Authors:** Katarzyna Nowakowska-Domagała, Marlena Podlecka, Patryk Stecz, Aleksandra Lewandowska, Kasper Sipowicz, Marcin Kosmalski, Tadeusz Pietras, Łukasz Mokros

**Affiliations:** 1 Institute of Psychology, Faculty of Educational Sciences, University of Lodz, Al. Rodzeństwa Scheiblerów 2, 90-128 Lodz, Poland; 2 Institute of Psychiatry and Neurology, Department of Neurosis, Personality and Eating Disorders, Sobieskiego Street 9, 02-957 Warsaw, Poland; 3 J. Babiński Specialist Psychiatric Health Care Team, Psychiatric Ward for Children, Aleksandrowska 159, 02-229 Lodz, Poland; 4 Department of Interdisciplinary Disability Studies, The Maria Grzegorzewska University in Warsaw, Szczęśliwicka 40, 02-353 Warsaw, Poland; 5 Department of Clinical Pharmacology, Medical University of Lodz, Kopcinskiego Street 22, 90-153 Lodz, Poland; 6 Institute of Psychiatry and Neurology, Second Department of Psychiatry, Sobieskiego Street 9, 02-957 Warsaw, Poland

**Keywords:** alcohol addiction, bipolarity features, depression, sleep quality, temperament

## Abstract

Clinical and epidemiological studies have demonstrated a relationship between alcohol addiction and mood disorders. Alcohol-dependent patients with depression tend to demonstrate clinically more severe manic symptoms, which complicates the process of diagnosis and therapy. However, the predictors indicating the risk of mood disorders in addicted patients remain unclear. The aim of the study was to examine the relationship between personal dispositions, bipolar traits, depth of addiction, quality of sleep, and depressive symptoms in alcohol-dependent men. The study group comprised 70 men (age M = 46.06, SD = 11.29) diagnosed with alcohol addiction. The participants completed a battery of questionnaires: BDI, HCL-32, PSQI, EPQ-R and MAST. The results were tested using Pearson’s correlation quotient and general linear model. The findings indicate that some of the studied patients are likely to have mood disorders of clinically significant severity. High neuroticism and poor sleep quality are independent predictors of depressive symptoms in alcohol-dependent patients. Among the components of sleep quality, problems with falling asleep and waking up at night appear to be most strongly associated with depressive symptoms. The intensity of depressive symptoms may relate to the intensity of certain bipolar features, such as risk-taking activity and irritability. High neuroticism and poor sleep quality are independent predictors of depressive symptoms in the studied group.

## 1. Introduction

Affective disorders and alcohol use disorder (AUD) are significant comorbidities [1,2,3,4]. Among people with AUD, as much as 50% meet the criteria of mood disorders [5,6]. Patients with such dual diagnosis are often described as clinically more severe, with complex courses of both disorders; as such, treatment can be more difficult than for patients with only one medical diagnosis [7,8]. The clinician must not only treat the addiction, typically the primary problem faced by the patient, but also the accompanying depression, especially when it is severe. In most cases, the patient is treated for one disorder before receiving treatment for the other [9]. This approach follows from the long-standing clinical perspective that treating the primary disorder can often resolve other problems, or that one disorder will be easier to treat if the other is in remission [10].

The joint presence of alcohol use disorder together with bipolar disorder correlates with longer duration of withdrawal from alcohol, increased severity of manic and depressive symptoms, increased suicide risk, higher financial costs, increased morbidity, poorer prognosis, and decreased overall degree of function [11,12]. It is also associated with a higher risk of relapse to alcohol dependence, treatment dropout, suicide attempt, and greater social and personal impairment, as well as poorer effects of antidepressant medication [2,3,9,13,14]. 

Perhaps the most commonly-cited explanations for the co-occurrence of alcohol-use disorder with mood disorders are causal models, suggesting that one condition creates an increased risk for the other [15]. Indeed, studies indicate that alcohol-use disorders increase the risk of depression [3,16,17,18,19] and manic episodes [20]. However, it has also been suggested that alcohol abuse may result from depressive symptoms as a form of self-medication [21,22] or from the more intense mood during hypomanic/manic episodes [23].

Poor sleep quality is common among patients with alcohol dependence during all stages, including active heavy use and early and prolonged abstinence [24]. The relationship between alcohol use and sleep is complex. In the non-alcoholic population, acute alcohol intake has sleep-promoting properties in the first half of the night [25,26]; hence, alcohol is often used as a somnolent, and this is used to justify initiating and continuing regular alcohol use [27]. Unfortunately, large quantities and/or long durations of alcohol use may worsen sleep quality, which has been observed in AUD patients [28]. In AUD, sleep disruptions typically manifest as altered sleep architecture, circadian rhythm abnormalities, increased sleep latency, insufficient sleep duration, excessive daytime sleepiness, and even profound insomnia [29,30]. Furthermore, sleep quality has been found to be an important predictor of risk of relapse [31], as well as the consequences of alcohol abuse, such as psychosocial impairment, decreased quality of life, and suicidal ideation [32,33,34]. In the general population, subjective sleep disturbances (interruptions in sleep) also increase the risk of depression [35].

A key role in the development of alcohol use is played by personal disposition [36]. Personality features are expressions of biologically-based systems which regulate individual sensitivities to positive and negative stimulation [37]. Thus, for example, extraverts seek positive affective stimuli and use alcohol for enhancement motives, whereas neurotics are sensitive to negative affective stimuli and use alcohol for coping motives [38]. Most of the previous studies have focused on three broad personality traits connected with problematic drinking patterns: impulsivity/novelty seeking, neuroticism/negative emotionality, and extraversion/reward dependence [39,40,41,42]. These personality characteristics are believed to serve as consistent predictors of AUD and mood disorders [2]. Inversely, alcohol abuse may predispose to changes in personality traits in adulthood, such as increased extraversion and decreased conscientiousness and emotional stability [43]. 

The main aim of this study was to examine the relationship between personal dispositions, bipolar traits, depth of addiction, quality of sleep, and depressive symptoms in alcohol-dependent men. We hypothesize that: 1. some of the patients studied are likely to have mood disorder symptoms of clinically significant severity; 2. there is an association between personality traits, quality of sleep, bipolarity, and depression among AUD patients; 3. personal dispositions and poor quality of sleep predict mood disturbances in alcohol dependence patients. 

## 2. Materials and Methods

### 2.1. Participants

The study was made in an outpatient center in Poland offering therapy to alcohol-dependent individuals. The study group consisted of 70 men diagnosed with alcohol dependence syndrome (F10.2) according to the ICD-10. The descriptive statistics for the studied sample are presented in Table 1. All patients were ethnically white Europeans. The research was carried out among consenting patients who had maintained alcohol abstinence for at least three weeks: this criterion negated the influence of alcohol abstinence syndrome symptoms on the study results. The exclusion criteria comprised the following: addictions to psychoactive substances other than alcohol and nicotine; serious mental disorders such as schizophrenia, depression, bipolar affective disorder, delirium tremens, alcohol-related psychosis, insomnia, or neurological disorders; somatic diseases such as malignant tumors, diabetes, hepatic cirrhosis, or ischemic heart disease, which may result in symptom overlap or measurement bias.

All patients were investigated during the third week of psychotherapy. Informed consent was obtained from the participants after the nature of the procedures had been fully explained. The raters were psychologists trained to use standardized questions and specific instructions. A total number of 63 patients entered the study: three patients were rejected from the statistical analysis because of incomplete questionnaires, and another four because of the co-occurrence of other addictions and mental illness.

### 2.2. Methods

The study uses Polish adaptations of the battery of well-known psychometric questionnaires, all of which are commonly used in clinical practice, psychological diagnosis, and for the purpose of scientific research.

The Polish version of the Eysenck Personality Questionnaire—Revised (EPQ-R), adapted by Brzozowski and Drwal [44], was used to assess personality in three main dimensions, according to Eysenck’s PEN theory: Psychoticism, Extraversion, and Neuroticism. The model also includes an additional Lie scale, measuring self-deception and impression adjustment. Although a newer version of the EPQ-R is present on the Polish market [45], the older version was chosen for the study due to its known suitable psychometric values for scientific research (Cronbach’s α was N-0.84, E-0.83, P-0.67, K-0.75) [44].

The severity of alcohol-related problems was assessed using the Michigan Alcoholism Screening Test (MAST) diagnostic questionnaire by Selzer well described in scientific works [46], adapted into Polish by Falicki et al. [47].

Depressive symptoms over the month prior to examination were assessed using a self-reported method–Beck Depression Index (BDI) [48,49]. The Polish adaptation was prepared by Zawadzki et al. [50], and Cronbach’s α was 0.95; in accordance with their suggestion, a cut-off value of 17 points was considered suggestive of clinically significant depressive symptoms for the Polish population.

To assess bipolar features, the Polish version of the Hypomania Checklist 32 (HCL-32) by [51], validated by Łojko et al. [52], including subscale assessed bipolar features connected with irritation and risk-taking- Subscale irr/ri-ta HCL-32 and subscale act/ela HCL-32—bipolar features connected with activity/elated and elevated mood was used. Cronbach’s alfa of Polish validation was 0.93, and a score of 14 or higher was considered as indicating bipolar features [52].

Sleep quality for the four weeks prior to the study was assessed using the Pittsburgh Sleep Quality Index (PSQI) which includes components: Subjective Sleep Quality, Sleep Duration, Sleep Latency, Habitual Sleep Efficiency, Use of Sleeping Medication, Sleep Disturbances (interruptions), and Daytime Dysfunction. The score is calculated on the basis of the respondent’s answers and each question is scored from 0 to 3 points, with Cronbach’s alpha of 0.82 [53]. A Polish translation is available via the website of the Centre of Sleep Medicine at Institute of Psychiatry and Neurology in Warsaw [54]. To the best of our knowledge, no validation has been published for the Polish population. The cutoff for poor sleep quality was regarded as five points, in accordance with the original version. Additionally, the questionnaire considering sociodemographic status designed by the authors.

Operationalization of Variables is Presented in Table 2.

### 2.3. Data Analyses

The data were analyzed with STATISTICA 12 PL (Kraków, Poland and Tulsa, OK, USA). Student’s *t*-test was used for comparisons between two independent groups. Pearson’s correlation coefficient was used to assess the relationship between two continuous variables, and general linear models were used to assess the effect of independent variables on the severity of depressive symptoms. The Holm-Bonferroni correction for multiple testing was utilized. Level of significance was adopted for alpha = 0.05. A post-hoc power analysis was performed with G*Power 3.1 software (Supplementary Materials).

## 3. Results

### 3.1. The Prevalence of Self-Reported Depressive Symptoms, Bipolar Features, and Poor Sleep Quality in a Study Group of Addict Men

It was found that 28.6% of the patients (N = 18) achieved a BDI score suggestive of clinically significant depressive symptoms (i.e., at least 17 points). In addition, 83% of the investigated group (N = 52) fulfilled the bipolarity criterion, based on an HCL-32 score of 14 points or more, and 47.6% of the patients (N = 30) reported poor sleep quality (at least 5 points in PSQI).

### 3.2. Comparison between Groups—Depression vs. No Depression

A result of 17 or more on the BDI was considered as an indicator of clinically-relevant self-described depressive symptoms; this was the cut-off point proposed by Zawadzki et al. [50] for the Polish population. In the study group, the mean BDI score was 11.94 (±8.97). Eighteen participants were classified as demonstrating depression based on their BDI score. The patients with BDI scores more than 17 achieved higher results in the EPQ-R Neuroticism scale (raw results and stens) and the PSQI Questionnaire and its dimensions (subjective sleep quality, sleep latency, sleep interruptions).

No statistically significant differences in HCL-32 score or subscale act/ela were found between patients with BDI < 17 and those with BDI > 17; however, significantly higher subscale irr/ri-ta scores were noted in patients with depressive symptoms (i.e., those with BDI > 17) (see Table 3).

### 3.3. Correlations

Selected correlation quotients confirm the findings given above. More precisely, statistically significant correlations were observed between depressive symptom intensity (BDI score) and neuroticism intensity (Neuroticism scale of EPQ-R score) and between BDI score and bipolar feature intensity (HCL-32), especially with regard to irr/ri-ta. No statistically significant correlation was found between BDI and MAST scores. More detailed results are shown in Table 4.

A significant positive correlation was found between HCL-32 irr/ri-ta score and the intensity of neuroticism assessed by the EPQ-R.

The intensity of depressive symptoms was also found to be associated with the intensity of bipolar features, such as risk activity taking and irritability, in the studied group; however, no such relationship was demonstrated for the “increased activity and elevated mood” component.

Of all the assessed components of sleep quality, it was found that problems falling asleep and waking up at night were most closely related to depressive symptoms. Hence, from a clinical perspective, these could be valuable topics for specialists to raise with patients with alcohol addiction to assess the risk of mood disorders.

### 3.4. Linear Regression Models Predicting the Severity of Depressive Symptoms

First, a logistic regression model was constructed to assess the influence of selected independent variables (age, years of addiction, EPQ-R subscales score, MAST score, HCl-32 subscales scores, PSQI score) on the intensity of depressive symptoms (BDI score) in the studied group. The results indicate that the only independent predictors were neuroticism and sleep quality, with a stronger relationship being demonstrated for sleep quality.

The coefficient of determination (R^2^) for Model 1 is 0.526: this model explained 53% of variance of the BDI score in the studied group. The calculated post-hoc power of the model was 1.0. More detailed results of the hierarchical model are given in Table 5.

Since sleep quality is such a relatively strong and independent predictor, it is possible to check the effect of the PSQI and its individual components on the severity of depressive symptoms independent of the other effects. All PSQI dimensions correlated with BDI scores in both the high BDI (>17) and the low BDI (<17) groups and in correlation analysis. However, when taking into account the simultaneous effect of all on BDI score, the only predictor of an increase in BDI score was found to be an increase in sleep disturbances (interruptions in sleep, i.e., waking up at night due to different factors).

The coefficient of determination (R^2^) for Model 2 is 0.361: this model explained 36% of the variance of the BDI score in the studied group. Calculated post-hoc power of the model was 0.999. More detailed results of the hierarchical model are given in Table 6.

## 4. Discussion

Our findings indicate that some of the alcohol-dependent patients (28.6%) are likely to experience clinically-significant depression. However, a large number of previous studies indicate a more significant likelihood of comorbidity of AUD and depression, reaching as high as 68% [5,55,56]. The disparity between our findings and those of previous studies may be due to our exclusion of patients with a diagnosis of mood disorders, and of those who failed to maintain alcohol abstinence for at least three weeks before the study.

The nature of depression may differ between individuals with alcohol dependence and those without; therefore, categorizing depression as independent (ID), i.e., in the course of mood disorders, or substance-induced (SID) could be a useful model for predicting the course of depression in alcohol use disorder (AUD) [57,58]. Substance-induced depression is thought to account for a substantial proportion of major depressive episodes among patients with AUD [59] and usually improves after two to three weeks of abstinence [60]; in contrast, independent depression does not necessarily remit with abstinence. Therefore, a period of abstinence of at least three weeks in patients without independent depression may result in total or partial withdrawal of depressive symptoms: this could explain the relatively low ratio of co-occurrence in the studied group.

Our findings also indicate that as many as 75% of patients with AUD fulfil the criterion of bipolarity. However, very little data has been gathered for the assessment of bipolarity in samples of alcohol-dependent patients. One of the few studies completed to date suggests that 6.5% of men with AUD have also suffered from at least one episode of mania [61]. Most of the previous research in this area has examined co-occurring alcohol abuse as a consequence of bipolar disorder [1,11]. However, alcohol-dependent patients currently in abstinence without diagnosis of bipolar disorder have been found to demonstrate high clinical severity of bipolar traits. This suggests that AUD withdrawal may prompt bipolarity symptoms. As such, this pathway of relationship requires further studies.

In the studied group, the intensity of depressive symptoms may be connected with the intensity of certain bipolar features, such as risk-taking activity and irritability. No such relationship has been demonstrated for the increased activity and elevated mood component. However, it should be noted that risk activity taking, and irritability are not independent of the structure of temperament, depth of addiction, duration of addiction, and quality of sleep. This subject therefore requires further study.

Our data also indicates that high neuroticism and poor sleep quality are independent predictors of depressive symptoms in alcohol-dependent patients. This is in line with previous findings that both of these indicators represent risk factors for depression [35,62,63,64]. More interestingly, however, from the point of view of our study, they are predictors of depression, regardless of the depth and duration of addiction; furthermore, they have been found to have no effect on the severity of depressive symptoms, independently of neuroticism and PSQI.

Insomnia is considered a risk factor of and a symptom of major depressive disorder, with the latter being reflected in the content of the diagnostic criteria [65,66]. Thus, the correlation between PSQI score and BDI score is not surprising in the studied group. However, testing the association between PSQI components and BDI score provided an answer to the question of which specific characteristics of sleep symptoms (including insomnia) might play the most important role in its link to depression among patients with addiction to alcohol. Among the studied components of sleep quality, problems with falling asleep and waking up at night were found to be most strongly associated with depressive symptoms in the study population. Hence, from a clinical perspective, these could be valuable topics for specialists to raise with patients with alcohol addiction to assess the risk of mood disorders.

There are some limitations inherent in the study. The lack of gender and racial diversity or relatively small sample size means that the sample may not be a truly representative, and the results of the study cannot be generalized across the entire alcohol addict men population. When analyzing the results obtained, further research is needed to establish to what extent results related to bipolar traits are really bipolar related or are more general personality traits (e.g., irritability or impulsivity). The same applies to traits that have previously been linked to alcohol (ab)use and sleep.

A limitation of this study was also the sleep quality assessment: this was based on self-report only, and as such, may include some subjectivity bias. However, previous studies have demonstrated that self-reported data is reliable [67]. In addition, the PSQI itself has good psychometric properties. In future studies, it may be worth considering expanding the scope of research by using actigraphy to validate physical activity and sleep/wake measurements. Due to the relatively rapid withdrawal of depressive symptoms after the onset of alcohol abstinence, its severity should be assessed both during an episode of drinking and during abstinence. Additionally, a comparison group of non-addicted individuals was not included. Yet, it should be considered the next research step to further answer the question whether risk factors delineated in this study are specific for the study group or reflect a link in a general population.

## 5. Conclusions

High neuroticism and poor sleep quality are independent predictors of depressive symptoms in alcohol-dependent patients. Among the components of sleep quality, problems with falling asleep and waking up at night appear to be most strongly associated with depressive symptoms. The intensity of depressive symptoms may relate to the intensity of certain bipolar features, such as risk-taking activity and irritability. High neuroticism and poor sleep quality are independent predictors of depressive symptoms in the studied group.

This is the first study to investigate the correlations between personal dispositions, mood disorders, and sleep quality among people with alcohol addiction. Although results are still preliminary and derived from a small population, they nevertheless have certain practical implications. Personality and sleep quality assessments may allow more accurate and faster identification of patients with alcohol dependency who may be at higher risk of mood disorders.

## Figures and Tables

**Table 1:** Demographic characteristics of the study group.

		Mean/SD or Number of Participants (%)
Age	Mean/ SD	46.06 11.29
	Min-Max	21–67
Age when addiction developed	Mean/ SD	30.95 10.59
	Min-Max	15–60
Duration of addiction	Mean/ SD	15.11 10.35
	Min-Max	1–40
Education	Basic	16 (25.4)
	Professional	20 (31.7)
	Average	21 (33.3)
	Higher	6 (9.5)
Marital status	single	12 (19.1)
	married	31 (49.2)
	divorced	19 (30.2)
	widower	1 (1.6)

M—mean value, SD—standard deviation, Min—minimal value, Max—maximal value.

**Table 2:** Presentation of dependent and independent variables included in the analysis, together with their indicators.

Variable	Index
Self-assessed depressive symptoms intensity	BDI score
Self-assessed bipolar features intensity	HCL-32 score
Self-assessed bipolar features connected with irritation and risk-taking	Subscale irr/ri-ta HCL-32
Self-assessed bipolar features connected with activity/elated and elevated mood	Subscale act/ela HCL-32
Depth of alcohol dependence (the severity of alcohol-related problems)	MAST score
Duration of alcohol dependence	Duration of alcohol dependence (in years) from sociodemographic questionnaire
Personality dimensions	Psychoticism EPQ-R score, Extraversion EPQ-R score, Neuroticism EPQ-R score, Lie scale EPQ-R score
Sleep quality	PSQI Score
Subjective quality of sleep	Component 1. PSQI score
Sleep latency	Component 2. PSQI score
Sleep duration	Component 3. PSQI score
Sleep efficiency	Component 4. PSQI score
Sleep disturbances (interruptions)	Component 5. PSQI score
Use of sleep medication	Component 6. PSQI score
Daytime dysfunction	Component 7. PSQI score

**Table 3:** Comparison of means (M) and standard deviations (SD) in EPQ-P, MAST, HCL-32 and PSQI questionnaires between patients with AD and depression (N = 18) and without depression (N = 45). This division was based on a BDI cut-off value of 17.

			Non-Depressed (N = 45)		Depressed (N = 18)		t	d	*p*
			M	SD	M	SD			
Age			46.27	11.45	45.56	11.21	0.22	0.062	0.99
Years of AD			13.89	9.92	18.17	11.05	−1.50	−0.417	0.98
**EPQ-R**	Neur	Raw	**10.80**	**6.09**	**17.44**	**3.99**	**−4.26**	**−1.192**	**0.02**
		Sten	**5.07**	**1.87**	**7.00**	**1.46**	**−3.91**	**−1.095**	**0.02**
	Extr	Raw	15.36	3.65	13.67	4.10	1.60	0.448	0,88
		Sten	6.07	1.44	5.67	1.50	0.99	0.276	0,99
	Psych	Raw	7.11	3.25	8.61	3.09	−1.67	−0.467	0,90
		Sten	4.36	2.02	5.39	1.75	−1.88	−0.525	0,70
	Lie	Raw	9.16	4.04	8.56	3.82	0.54	0.152	0,99
		Sten	5.68	1.90	5.33	1.64	0.68	0.190	0,99
MAST score			**34.22**	**12.87**	**44.17**	**15.63**	**−2.60**	**−0.726**	**0.14**
**HCL−32**	total		15.59	5.21	17.67	5.36	−1.41	−0.395	0.96
	act/ela		9.59	3.43	9.44	4.53	0.14	0.039	0.89
	irr/ri-ta		**2.18**	**1.85**	**3.89**	**2.40**	**−3.02**	**−0.846**	**0.02**
**PSQI**	total		**5.40**	**2.41**	**10.72**	**5.46**	**−5.40**	**−1.505**	**0.02**
	Subjective sleep quality		**0.82**	**0.53**	**1.56**	**0.92**	**−3.95**	**−1.102**	**0.02**
	Sleep latency		**1.00**	**0.85**	**1.72**	**0.83**	**−3.06**	**−0.854**	**0.02**
	Sleep duration		**0.67**	**0.85**	**1.39**	**1.04**	**−2.85**	**−0.796**	**0.13**
	Habitual sleep efficiency		**0.44**	**0.76**	**1.11**	**1.23**	**−2.62**	**−0.730**	**0.12**
	Sleep interruptions		**1.07**	**0.54**	**1.83**	**0.92**	**−4.11**	**−1.146**	**0.02**
	Use of sleeping meds		**0.47**	**0.99**	**1.33**	**1.41**	**−2.76**	**−0.770**	**0.11**
	Daytime dysfunction		**0.93**	**0.62**	**1.78**	**1.11**	**−3.84**	**−1.071**	**0.02**

t—statistics in the test; d—size of effect (Cohen’s d); *p*—probability in the statistical test (after Holm-Bonferroni correction for multiple testing); statistically significant results are bolded; EPQ-R—Eysenck Personality Questionnaire—Revised; Neur—Neuroticism; Extr—Extraversion; Psych—Psychoticism; MAST—Michigan Alcoholism Screening Test; HCL-32—Hypomania Checklist; act/ela—active/elated; irr/ri-ta—irritable/risk-taking; PSQI—Pittsburgh Sleep Quality Inventory.

**Table 4:** Correlation matrix between continuous variables used in the analysis.

			Age	A	B	C	D	E	F	G	H	I	J	K	L	M	N	O
**A**	Years of AD		0.551 *															
**B**	EPQ-R Neur	Raw	−0.026	0.252														
**C**		Sten	−0.052	0.174	0.957 *													
**D**	EPQ-R Extr	Raw	−0.152	−0.145	−0.174	−0.108												
**E**		Sten	−0.150	−0.147	−0.220	−0.149	0.949 *											
**F**	EPQ-R Psych	Raw	0.132	0.077	0.198	0.183	−0.087	−0.055										
**G**		Sten	0.121	0.093	0.201	0.191	−0.084	−0.049	0.971 *									
**H**	EPQ-R Lie	Raw	−0.173	−0.260	−0.066	−0.113	−0.013	−0.072	−0.155	−0.250								
**I**		Sten	−0.150	−0.263	−0.056	−0.109	−0.013	−0.080	−0.154	−0.242	0.985 *							
**J**	MAST score		0.007	0.415	0.230	0.167	−0.137	−0.113	0.068	0.086	−0.063	−0.074						
**K**	HCL−32	total	−0.071	0.080	0.274	0.257	0.213	0.149	0.020	−0.005	0.093	0.091	0.124					
**L**		act/ela	−0.087	−0.044	−0.004	0.009	0.163	0.103	−0.210	−0.241	0.205	0.191	0.113	0.795 *				
**M**		irr/ri-ta	−0.095	0.036	0.345	0.315	0.152	0.163	0.359	0.362	−0.207	−0.194	0.038	0.455 *	−0.053			
**N**	BDI score		−0.015	0.221	0.622 *	0.602 *	−0.136	−0.120	0.143	0.158	−0.079	−0.105	0.243	0.262	0.017	0.412 *		
**O**	PSQI	total	−0.186	0.023	0.421 *	0.417 *	0.113	0.106	0.208	0.214	0.005	−0.012	0.299	0.317	0.138	0.380	0.634 *	
		1.	−0.207	0.059	0.326	0.322	0.145	0.107	0.175	0.206	−0.011	−0.026	0.250	0.335	0.153	0.358	0.485 *	0.844 *
		2.	−0.137	0.039	0.219	0.216	−0.061	−0.083	0.222	0.238	0.138	0.123	0.075	0.121	0.039	0.094	0.398	0.586 *
		3.	−0.093	−0.024	0.251	0.252	0.089	0.083	0.131	0.142	−0.079	−0.059	0.202	0.149	0.012	0.273	0.408 *	0.656 *
		4.	−0.141	−0.033	0.176	0.224	0.017	0.040	0.316	0.323	−0.074	−0.086	0.117	0.135	0.069	0.223	0.318	0.671 *
		5.	−0.148	−0.072	0.373	0.383	0.203	0.219	0.042	0.024	−0.001	−0.012	0.016	0.217	−0.018	0.410 *	0.578 *	0.717 *
		6.	0.046	0.160	0.323	0.256	−0.045	−0.015	0.067	0.038	0.088	0.053	**0.422**	0.229	0.175	0.140	0.357	0.566 *
		7.	−0.278	−0.072	0.336	0.352	0.259	0.211	0.041	0.064	−0.068	−0.073	0.239	0.334	0.192	0.379	0.513 *	0.763 *

*—*p* < 0.05 after Holm-Bonferroni correction; statistically significant results are bolded. EPQ-R—Eysenck Personality Questionnaire—Revised; Neur—Neuroticism; Extr—Extraversion; Psych—Psychoticism; MAST—Michigan Alcoholism Screening Test; HCL-32—Hypomania Checklist; act/ela—active/elated; irr/ri-ta—irritable/risk-taking; BDI—Beck Depression Inventory; PSQI—Pittsburgh Sleep Quality Inventory, total score; 1.—Subjective sleep quality; 2.—Sleep latency; 3.—Sleep duration; 4.—Habitual sleep efficiency; 5.—Sleep disturbances (interruptions); 6.—Use of sleeping meds; 7.—Daytime dysfunction.

**Table 5:** Model 1.

		B	B 95% CI	Beta	Beta 95% CI		t		*p*
Age		0.029	−0.156	0.214	0.037	−0.197	0.272	0.318	0.752
Years of AD		0.084	−0.143	0.311	0.096	−0.164	0.357	0.742	0.462
EPQ-R	Neur	**0.480**	**0.170**	**0.789**	**0.339**	**0.120**	**0.557**	**3.116**	**0.003**
	Extr	−0.348	−0.804	0.108	−0.149	−0.345	0.046	−1.532	0.132
	Psych	−0.321	−0.877	0.235	−0.117	−0.320	0.086	−1.160	0.251
	Lie	−0.018	−0.456	0.420	−0.008	−0.202	0.186	−0.084	0.933
MAST score		−0.023	−0.161	0.115	−0.036	−0.255	0.182	−0.335	0.739
HCL-32	act/ela	−0.064	−0.524	0.396	−0.027	−0.219	0.165	−0.280	0.781
	irr/ri-ta	0.732	−0.184	1.648	0.174	−0.044	0.392	1.606	0.115
PSQI score		**1.019**	**0.551**	**1.486**	**0.485**	**0.263**	**0.708**	**4.377**	**0.000**
Intercept		3.583	−10.597	17.764				0.508	0.614

t—statistics in the test; *p*—probability in the statistical test; 95% CI—95% Confidence interval for the mean value; statistically significant results are bolded; EPQ-R—Eysenck Personality Questionnaire—Revised; Neur—Neuroticism; Extr—Extraversion; Psych—Psychoticism; MAST—Michigan Alcoholism Screening Test; HCL-32—Hypomania Checklist; act/ela—active/elated; irr/ri-ta—irritable/risk-taking; PSQI—Pittsburgh Sleep Quality Inventory.

**Table 6:** Model 2, including PSQI components only.

	B	B 95% CI		Beta	Beta 95% CI		t	*p*
1. Subjective sleep quality	0.505	−3.439	4.449	0.042	−0.284	0.367	0.257	0.799
2. Sleep latency	1.400	−1.060	3.860	0.141	−0.106	0.388	1.141	0.259
3. Sleep duration	1.093	−1.592	3.778	0.117	−0.170	0.404	0.816	0.418
4. Habitual sleep efficiency	−0.528	−2.964	1.908	−0.056	−0.316	0.203	−0.434	0.666
5. Sleep interruptions	**3.816**	**0.569**	**7.063**	**0.319**	**0.048**	**0.591**	**2.355**	**0.022**
6. Use of sleeping meds	1.294	−0.452	3.041	0.171	−0.060	0.401	1.485	0.143
7. Daytime dysfunction	1.762	−1.070	4.593	0.171	−0.104	0.446	1.247	0.218
Intercept	1.207	−2.689	5.103				0.621	0.537

t—statistics in the test; *p*—probability in the statistical test; 95% CI—95% Confidence interval for the mean value; statistically significant results are bolded; PSQI—Pittsburgh Sleep Quality Inventory components.

## Data Availability

The data that support the findings of this study are available from the corresponding author, [KND], upon reasonable request.

## Supplementary Materials

The following supporting information can be downloaded at: https://www.mdpi.com/article/10.3390/ijerph20054072/s1, Table S1. Comparison of means (M) and standard deviations (SD) in EPQ-P, MAST, HCL-32 and PSQI questionnaires between patients with AD and depression (N = 18) and without depression (N = 45). This division was based on a BDI cut-off value of 17. Presented with results of post-hoc power analysis (1 − β). Table S2. Results of a post-hoc power analysis (1 − β) of the calculated Pearson correlation quotients shown in Table 2.
