# Supplementary material for: The Relationship between Selected Factors (Temperament, Bipolar Traits, Sleep Quality, Severity of Addiction) and Depressive Symptoms in Alcohol-Dependent Men
Source: Int J Environ Res Public Health. 2023 Feb 24;20(5):4072. doi: 10.3390/ijerph20054072 (PMC10001749; doi:10.3390/ijerph20054072)
Supplement: Supplementary file 1 [file ijerph-20-04072-s001.zip › ijerph-2206822-supplementary.pdf]

**Supplementary Table S1.** Comparison of means (M) and standard deviations (SD) in EPQ-P, MAST, HCL-32 and PSQI questionnaires between patients with AD and depression (N = 18) and without depression (N = 45). This division was based on a BDI cut-off value of 17. Presented with results of post-hoc power analysis (1-β).

| | | | Non-depressed (N=45) | | Depressed (N=18) | | t | df | p | d | 1-β |
|---|---|---|---|---|---|---|---|---|---|---|---|
| | | | M | SD | M | SD | | | | | |
| Age | | | 46.27 | 11.45 | 45.56 | 11.21 | 0.22 | 61 | 1.640 | 0.062 | 0.056 |
| Years of AD | | | 13.89 | 9.92 | 18.17 | 11.05 | -1.50 | 61 | 0.980 | -0.417 | 0.313 |
| EPQ-R | Neur | Raw | 10.80 | 6.09 | 17.44 | 3.99 | -4.26 | 60 | 0.022 | -1.192 | 0.988 |
| | | Sten | 5.07 | 1.87 | 7.00 | 1.46 | -3.91 | 60 | 0.021 | -1.095 | 0.972 |
| | Extr | Raw | 15.36 | 3.65 | 13.67 | 4.10 | 1.60 | 60 | 0.880 | 0.448 | 0.353 |
| | | Sten | 6.07 | 1.44 | 5.67 | 1.50 | 0.99 | 60 | 1.650 | 0.276 | 0.164 |
| | Psych | Raw | 7.11 | 3.25 | 8.61 | 3.09 | -1.67 | 60 | 0.900 | -0.467 | 0.378 |
| | | Sten | 4.36 | 2.02 | 5.39 | 1.75 | -1.88 | 60 | 0.700 | -0.525 | 0.457 |
| | Lie | Raw | 9.16 | 4.04 | 8.56 | 3.82 | 0.54 | 60 | 1.770 | 0.152 | 0.084 |
| | | Sten | 5.68 | 1.90 | 5.33 | 1.64 | 0.68 | 60 | 2.000 | 0.190 | 0.103 |
| MAST score | | | 34.22 | 12.87 | 44.17 | 15.63 | -2.60 | 61 | 0.140 | -0.726 | 0.726 |
| HCL-32 | | total | 15.59 | 5.21 | 17.67 | 5.36 | -1.41 | 60 | 0.960 | -0.395 | 0.286 |
| | | act/ela | 9.59 | 3.43 | 9.44 | 4.53 | 0.14 | 60 | 0.890 | 0.039 | 0.052 |
| | | irr/ri-ta | 2.18 | 1.85 | 3.89 | 2.40 | -3.02 | 60 | 0.020 | -0.846 | 0.847 |
| PSQI | | total | 5.40 | 2.41 | 10.72 | 5.46 | -5.40 | 61 | 0.019 | -1.505 | 1.000 |
| | | 1. Subjective sleep quality | 0.82 | 0.53 | 1.56 | 0.92 | -3.95 | 61 | 0.018 | -1.102 | 0.973 |
| | | 2. Sleep latency | 1.00 | 0.85 | 1.72 | 0.83 | -3.06 | 61 | 0.017 | -0.854 | 0.854 |
| | | 3. Sleep duration | 0.67 | 0.85 | 1.39 | 1.04 | -2.85 | 61 | 0.130 | -0.796 | 0.802 |
| | | 4. Habitual sleep efficiency | 0.44 | 0.76 | 1.11 | 1.23 | -2.62 | 61 | 0.120 | -0.730 | 0.731 |
| | | 5. Sleep disturbances | 1.07 | 0.54 | 1.83 | 0.92 | -4.11 | 61 | 0.016 | -1.146 | 0.981 |
| | | 6. Use of sleeping meds | 0.47 | 0.99 | 1.33 | 1.41 | -2.76 | 61 | 0.110 | -0.770 | 0.776 |
| | | 7. Daytime dysfunction | 0.93 | 0.62 | 1.78 | 1.11 | -3.84 | 61 | 0.015 | -1.071 | 0.966 |

t – statistics in the test; d – size of effect (Cohen's d), df – degrees of freedom, 1-β – post-hoc power, p – probability in the statistical test (after Holm-Bonferroni correction for multiple testing), 95%CI – 95% Confidence interval for the mean value, EPQ-R – Eysenck Personality Questionnaire – Revised; Neur – Neuroticism; Extr – Extraversion; Psych – Psychoticism; MAST - Michigan Alcoholism Screening Test; HCL-32 – Hypomania Checklist; act/ela - active/ elated; irr/ri-ta - irritable/risk-taking; PSQI – Pittsburgh Sleep Quality Inventory.

**Supplementary Table S2.** Results of a post-hoc power analysis (1-β) of the calculated Pearson correlation quotients shown in Table 2.

| | | | | Age | A | B | C | D | E | F | G | H | I | J | K | L | M | N | O |
|---|---|---|---|---|---|---|---|---|---|---|---|---|---|---|---|---|---|---|---|
| A | Years of AD | | | 0.999 | | | | | | | | | | | | | | | |
| B | EPQ-R | Neur | Raw | 0.055 | 0.530 | | | | | | | | | | | | | | |
| C | | | Sten | 0.069 | 0.282 | 1.000 | | | | | | | | | | | | | |
| D | | Extr | Raw | 0.224 | 0.208 | 0.282 | 0.136 | | | | | | | | | | | | |
| E | | | Sten | 0.220 | 0.213 | 0.422 | 0.218 | 1.000 | | | | | | | | | | | |
| F | | Psych | Raw | 0.180 | 0.093 | 0.351 | 0.307 | 0.105 | 0.071 | | | | | | | | | | |
| G | | | Sten | 0.159 | 0.113 | 0.361 | 0.330 | 0.101 | 0.067 | 1.000 | | | | | | | | | |
| H | | Lie | Raw | 0.279 | 0.557 | 0.081 | 0.144 | 0.051 | 0.087 | 0.232 | 0.523 | | | | | | | | |
| I | | | Sten | 0.221 | 0.567 | 0.072 | 0.144 | 0.051 | 0.096 | 0.230 | 0.495 | 1.000 | | | | | | | |
| J | MAST score | | | 0.050 | 0.946 | 0.455 | 0.263 | 0.191 | 0.144 | 0.083 | 0.104 | 0.078 | 0.089 | | | | | | |
| K | HCL-32 | | total | 0.086 | 0.096 | 0.605 | 0.547 | 0.399 | 0.218 | 0.053 | 0.050 | 0.113 | 0.110 | 0.164 | | | | | |
| L | | | act/ela | 0.105 | 0.064 | 0.050 | 0.051 | 0.252 | 0.128 | 0.389 | 0.492 | 0.373 | 0.330 | 0.144 | 1.000 | | | | |
| M | | | irr/ri-ta | 0.116 | 0.059 | 0.819 | 0.737 | 0.225 | 0.252 | 0.852 | 0.858 | 0.380 | 0.339 | 0.060 | 0.979 | 0.070 | | | |
| N | BDI score | | | 0.052 | 0.425 | 1.000 | 1.000 | 0.189 | 0.157 | 0.204 | 0.240 | 0.095 | 0.131 | 0.499 | 0.564 | 0.052 | 0.942 | | |
| O | PSQI | | total | 0.316 | 0.054 | 0.952 | 0.948 | 0.144 | 0.132 | 0.383 | 0.402 | 0.050 | 0.051 | 0.687 | 0.742 | 0.193 | 0.894 | 1.000 | |
| | | | 1. | 0.380 | 0.075 | 0.768 | 0.757 | 0.208 | 0.134 | 0.284 | 0.376 | 0.051 | 0.055 | 0.523 | 0.793 | 0.227 | 0.850 | 0.991 | 1.000 |
| | | | 2. | 0.191 | 0.061 | 0.418 | 0.408 | 0.077 | 0.100 | 0.428 | 0.482 | 0.193 | 0.162 | 0.090 | 0.159 | 0.061 | 0.114 | 0.923 | 1.000 |
| | | | 3. | 0.113 | 0.054 | 0.526 | 0.530 | 0.107 | 0.100 | 0.178 | 0.202 | 0.095 | 0.075 | 0.364 | 0.218 | 0.051 | 0.601 | 0.937 | 1.000 |
| | | | 4. | 0.199 | 0.058 | 0.287 | 0.435 | 0.052 | 0.061 | 0.740 | 0.760 | 0.089 | 0.104 | 0.151 | 0.187 | 0.084 | 0.431 | 0.745 | 1.000 |
| | | | 5. | 0.215 | 0.087 | 0.881 | 0.899 | 0.367 | 0.418 | 0.062 | 0.054 | 0.050 | 0.051 | 0.052 | 0.412 | 0.052 | 0.940 | 1.000 | 1.000 |
| | | | 6. | 0.065 | 0.245 | 0.760 | 0.543 | 0.064 | 0.052 | 0.082 | 0.060 | 0.106 | 0.070 | 0.953 | 0.451 | 0.284 | 0.197 | 0.847 | 1.000 |
| | | | 7. | 0.618 | 0.087 | 0.796 | 0.836 | 0.554 | 0.392 | 0.062 | 0.079 | 0.083 | 0.088 | 0.485 | 0.790 | 0.333 | 0.892 | 0.997 | 1.000 |

EPQ-R – Eysenck Personality Questionnaire – Revised; Neur – Neuroticism; Extr – Extraversion; Psych – Psychoticism; MAST - Michigan Alcoholism Screening Test; HCL-32 – Hypomania Checklist; act/ela - active/ elated; irr/ri-ta - irritable/risk-taking; BDI – Beck Depression Inventory; PSQI – Pittsburgh Sleep Quality Inventory, total score; 1.- Subjective sleep quality; 2. -Sleep latency; 3.- Sleep duration; 4.-Habitual sleep efficiency; 5.-Sleep disturbances (interruptions); 6.- Use of sleeping meds; 7.-Daytime dysfunction.
